# Risk factors associated with esophageal cancers, diagnosed at tertiary level in Afghanistan: a descriptive cross-sectional study

**DOI:** 10.1186/s12885-022-10228-9

**Published:** 2022-10-31

**Authors:** Ramin Saadaat, Jamshid Abdul-Ghafar, Ahmed Nasir Hanifi, Saifullah Khalid, Abdul Latif Khairy, Abdul Sami Ibrahimkhil, Haider Ali Malakzai, Esmatullah Esmat, Mujtaba Haidari, Nasrin Hussaini, Najla Nasir, Sarah Noor, Ahmed Maseh Haidary

**Affiliations:** 1 Department of Pathology and Clinical Laboratory, French Medical Institute for Mother and Children (FMIC), Kabul, Afghanistan; 2 Central Public Health Laboratory, Ministry of Public Health, Kabul, Afghanistan; 3 Department of Radiology, Jumhoriat Hospital, Kabul, Afghanistan; 4 Gastro-enterology unit, Department of Medicine, Rabia Balkhi Hospital, Kabul, Afghanistan; 5 Department of Oncology, Ali Abad Hospital, Kabul, Afghanistan

**Keywords:** Esophageal cancer, Risk factors, Socioeconomic, Low education

## Abstract

**Background:**

Worldwide, esophageal cancer (EC) is a common cancer in term of incidence and mortality and is the 4th common cancer in Afghanistan. Current study aimed to evaluate the profile of risk factors for EC among patients diagnosed at tertiary level in Afghanistan.

**Methodology:**

A descriptive cross-sectional study was carried out between January 2019 up to February 2021 including all esophageal cancers diagnosed at pathology department of French Medical Institute for Mothers and Children, Afghanistan.

**Result:**

240 diagnosed cases were analyzed, in which 59.40% of squamous cell carcinoma and 41.07% Adenocarcinoma. Both histopathological type of were predominantly diagnosed in males. The majority of the patients were residents of rural areas. More than 80% of the patients were illiterate with only less than 2% completing higher education. Majority of the patients were laborers and farmers while less than 10% were employed. According to income assessment, more than 80% were from low-income household, the rest from middle-income and none from high-income family. Oral snuff consumption was noted in 33.9% of squamous cell carcinoma patients and 40% adenocarcinoma patients whereas, family history of esophageal cancer was observed in 37.8% and 36.7% in both types of carcinomas, respectively. More than 60% of both types of carcinomas patients were hot tea drinkers.

**Conclusion:**

Current study demonstrated that most patients diagnosed with esophageal cancers were male, uneducated, belongs to low-income groups, lives in rural areas. These findings suggest distribution of esophageal cancer in specific socioeconomic groups, clearly demonstrating the need further analytical study.

**Supplementary Information:**

The online version contains supplementary material available at 10.1186/s12885-022-10228-9.

## Introduction

Globally, esophageal cancer (EC) is the 7th most common cancer in term of incidence and 6th in term of mortality. In 2018, it was estimated that one in every 20-cancer related deaths belonged to EC. The incidence is widely variable in different parts of the world [1]. In Afghanistan, EC is the fourth-most common cancer in both the sexes, and fifth common cancer in women, as reported by GLOBOCAN 2018 [2, 3]. The incidence rate of the EC may vary by up to 20-fold depending on regions of the world [4].

EC arises from the surface lining epithelial cells of esophagus. Squamous cell carcinoma (SCC) and adenocarcinoma (AC) are the two most common types of EC, while Less than 1% are sarcomas or small cell carcinomas, with very rarely reported incidence of esophageal lymphoma, melanoma, or carcinoid [3]. The main risk factors for EC in Asian belt till now have not been understood very well [5]. Poor nutritional status, low intake of fruits and vegetables, low socioeconomic status, smoking and excessive alcohol consumption are known as putative risk factors [4, 6]. Association between EC and low socio-economic status have been reported in a study conducted in Kashmir that was supported by another study in Iran, demonstrating strong relationship between EC and low socio-economic status, low education level and residence in rural areas of the country [7, 8]. In Afghanistan, no reported data is available to study the risk factors for EC and therefore the current study was conducted to demonstrate the frequency of various risk factors for EC at tertiary level in the country. A previous study conducted in northern Afghanistan was based on endoscopic examination findings for diagnosed of esophageal cancer without consideration of the histopathological features [9].

## Materials and methods

### Patients and factors evaluated

The project was approved by ethical review committee of the French Medical Institute for Mothers and Children (FMIC). This was a descriptive cross-sectional study, conducted between January 2019 up to February 2021, for EC cases diagnosed in the department of Pathology and clinical laboratory of FMIC. All the cases were biopsied during endoscopy and sent to histopathology section for confirmative diagnosis. The biopsies were submitted in 10% Formalin solution prior to further processing. Paraffin embedded issue sections were stained with hematoxylin and eosin (H&E) and then the slides were examined under microscope for diagnosis, histologic sub-classification and determination of the level of differentiation of the carcinoma. Prior to interview, all the patients were asked if they want to be part of our study, and informed consent was acquired from those who accepted to be part of our study. A self-structured questionnaire included age, gender, ethnicity group, residence, education level and income were asked from all patients who were diagnosed with EC. Ethnicity groups were segregated into four large groups that are Pashtun, Tajik, Uzbek-Turkmen and Hazara, while small ethnic groups were labeled as others. Residence area was labelled as rural or urban and province of the country, which furtherly divided the data into seven health zone of the country. Education level was categorized as illiterate, primary school, high school level, more than high school and university level. Income was also categorized as low, middle and high. Patients who were labor worker, with no business property, car and own house were considered as low income. Patients who had job, with own house but no additional properties or business were labelled as middle income and patients who had almost permanent high salary job, own houses, cars or additional properties were considered in high income groups.

### Statistical analysis

Means and Standard Deviations were calculated for continuous variables while frequency and proportions were calculated for categorical variables. The analyses were performed with the SPSS, version 25.

### Results

Current study included 240 EC cases, of which 75% were SCC and 25% were AC, as shown in Table 1. Majority of the cases were diagnosed in male, constituting 71% and 78,3%, respectively for SCC and AC, shown in Table 1. The mean age at diagnosis for SCC was 59.74 ± 9.02 y and for AC was 60.15 ± 9.96 y, also shown in Table 1. The lowest age of occurrence was 35y for both type of the carcinomas. Most of the SCC cases (40%) and AC cases (38.3%) were diagnosed between the age of 60–69 years, as shown in Table 1.

**Table 1:** Socio-demography and family history of the study population

Variables		SCC = 180	AC = 60	
		Number (%)	Number (%)	
**Age group (year)**				
	30–39	1 (0.6%)	1 (1.7%)	
	40–49	19 (10.6%)	7 (11.7%)	
	50–59	59 (32.8%)	15 (25. %)	
	60–69	72(40%)	23 (38.3%)	
	> 70	29 (16.10%)	14 (23.30%)	
Mean age (SD*)		59.74 ± 9.02 Y	60.15 ± 9.96 Y	
**Gender**	Male	128 (71.1%)	47 (78.3%)	
	Female	52 (28.9%)	13 (21.7%)	
**Ethnicity Group**	Pashton	68 (37.8%)	24 (40%)	
	Tajik	57 (31.7%)	19 (31.7%)	
	Uzbek-Turkmen	28 (15.6%)	7 (11.7%)	
	Hazara	24 (13.3%)	8 (13.3%)	
	Others	3 (1.7%)	2 (%3.3)	
**Education level**	Illiterate	147 (81.7%)	50 (83.3%)	
	Primary school	31 (17.2%)	9 (15%)	
	secondary/high school	2 (1.1%)	1(1.7%)	
**Residency**	Rural	162(90.0%)	52 (86.7%)	
	Urban	18 (10.0%)	8 (13.3%)	
**Country zone**	Center	49 (27.2%)	19 (31.7%)	
	North-East	38 (21.1%)	15 (25.0%)	
	North	31 (17.2%)	8 (13.3%)	
	South-East	24 (13.3%)	6 (10.0%)	
	West	18 (10%)	5 (8.3%)	
	South	11 (6.1%)	4 (6.7%)	
	East	9 (5.0%)	3 (5.0%)	
**Occupation**	Farmer	76(42.2%)	46 (60.6%)	
	House wife	52 (28.9%)	9(15%)	
	Non-employee	44 (24.4%)	10 (16.7%)	
	Employee	8 (4.4%)	5 (8.3%)	
**Income**	Low	151 (83.9%)	51 (85%)	
	Middle	29 (16.1%)	9 (15%)	
	High	0 (0%)	0 (0%)	
**Exercise**	Never	175 (97.2%%)	57 (95.0%)	
	Ever	5 (2.8%)	3 (5%)	
**Family history of EC**	No	112 (62.2%)	38 (63.3%)	
	Yes	68 (37.8%)	22 (36.7%)	
**Family history relationship**	Parents	23 (54.8%)	4 (36.4%)	
	Brothers and sisters	19 (45.2%)	7 (63.3%)	

*Standard deviation

Shown in Table 1, majority of the patients were living in rural areas (SCC 93.2% and AC 86.7%) and the remaining were residents of urban regions of the country. Similarly, considering regional distribution, most of the patient were from center zone of the country, constituting 31.7% for SCC and 27.2% for AC. The second most common country region was North-East constituting 21.1% for SCC and 25% for AC cases. Majority of the patients, i.e., 81.7% of SCC and 83.1.7% of AC case were illiterate, only 1.1% of SCC and 1.7% of AC cases had completed high-school educations and the rest were having the education of primary school.

As shown in Table 1, considering the disease distribution in various ethnic groups of the country, both, the SCC and AC were most common in Pashtun, constituting 37.8% of SCC and 40% of the cases respectively, followed by Tajik, Uzbek and Hazara. Large number of the patients belonged to the low-income families (83.9% of SCC) and (85% of the AC), the rest were having middle income, while none of patients belonged to high income families, also shown in Table 1. Similarly, shown in Tables 2 16.7% of the SCC patients and 15% of AC patients were cigarette smokers. 33.9% of SCC and 40% of AC cases were oral snuff users. Nearly all patients were non-alcoholic in our study, as only 4 out of 240 patients had very occasionally experienced alcohol drinking during their lifetime. According to tea drinking habit, majority of SCC and AC patient had the habit of drinking tea at higher temperature 63.8% and 61.7%, respectively.

**Table 2:** Hazardous and habits characteristic of patients

Variables		SCC = 180	AC = 60	
		Number (%)	Number (%)	
**Smoking**	No	150 (83.3%)	51 (85%)	
	Yes	30 (16.7%)	9 (15%)	
**Smoking duration**	Up to 10 year	13 (50%)	5 (38.4%)	
	More than 10 year	13 (50%)	13 (61.6%)	
**Oral snuff using**	No	119 (66.1%)	36 (60%)	
	Yes	61 (33.9%)	24 (40%)	
**Duration of snuff using**	Up 10 years	5 (8.9%)	4 (13.8%)	
	More than 10 years	51 (91.1%)	25 (86.2%)	
**Alcohol drinking**	No	178 (98.9%)	58 (96.7%)	
	Yes	2 (1.1%)	2 (3.3%)	
**Hot tea drinking habit**	No	65 (36.1%)	23 (38.3%)	
	Yes	115 (63.8%)	37 (61.7%)	

Of the male patients in our study, 42.2% of SCC and 60.6% of AC cases were diagnosed in farmers while all female patients in both carcinoma cases were house wives. Non-employed including laborers and simple workers constituted 24% of the SCC cases and 16.7% of AC cases and only 4.4% and 8.3% of the SCC and AC patients respectively were having office work or own business. In addition, near all patients of both group of carcinomas (97.2% of SCC and 95% of AC) were not doing regular exercise. In our study, 37.8% of SCC patients and 36.7% 0f AC patient had history of EC in first degree family members.

## Discussion

EC mostly involves older people and the risk rises with age, with an average age of 67 year at the time of diagnosis [10]. In our study age group “60 years and above” was the most common age group for both SCC & AC of the esophagus. Studies conducted in various neighboring countries reported similar findings [11–13]. The EC most commonly affects male than female population. The incidence of AC was reported to be 6–10 times more in male as compared to female patients, while the incidence of esophageal SCC was reported to be 2–3 times more in male as compared to female patients [14]. The current study also demonstrated that majority of the EC cases were diagnosed in males. The higher incidences of EC in males maybe related to their higher exposure to possible risk factors such as smoking, snuff dipping and exposure to agricultural products with EC cases mostly reported in farmers. The predominant histologic type of the EC in our study was the SCC which comprised (75%) followed by the AC that comprised of (25%). World-wide, SCC and AC were most common cancers of esophagus and constituted 95% of all EC cases. However, globally SCC is the predominant type of EC [15]. It has been also illustrated that SCC was more prevalent in under-developed countries and AC was prevalent in developed countries [16, 17]. The current study demonstrated that most of the patients with EC belonged to low-income families (more than 80%) and up to 80% were uneducated. Low socio-economic status and low education level were related the increases of the EC incidence, as per findings by researches conducted elsewhere. Studies in china and Iran reported strong association between low socio-economic status and an increased risk of EC [18–20]. The increased risk of EC in low socioeconomic setting was also reported in developed and rich countries with newly growing economies [21, 22].

A study in India indicated that 30.91% of EC patients were illiterates, 73.91% patients belonged to lower socio-economic status [23]. The association between mortality due to EC and lower education has also been reported [24].

Nearly all patients of EC in current study were living in rural areas of the country. These findings were similar to data from studies conducted in other countries that demonstrated higher incidence with significant association of developing EC and rural residence [7, 25]. In our study, the majority of the male patients with EC were farmers, constituting 48% of SCC and 55% of the AC group. Many other studies have reported high incidence of EC in farmers. A Study reported farmers to have increased risk for EC [26]. Study conducted in Brazil also reported that EC was more prevalent in farmers in high prevalent area [27]. A study from Iran reveals that majority of men with EC were farmers by occupation [20]. The work environment in agriculture is complex, with many potential hazardous exposures, such as pesticides, herbicides, fertilizers, dusts, zoonotic microbes, and sunlight [28].

The EC cases in the present study were mostly from center zone of the country and more common in Pashtun ethnic group following by Tajiks. Apart from that there was no ethnic predilection in other parts of the country, probably because the Pashtun and Tajik mainly populate the center zone, which is the most populated region in the country.

In our study, 33% of SCC and 40% of AC patient were oral snuff users. In majority of the studies conducted on EC, snuff consumption was documented to be the most common risk factor for development of EC. A multinational study conducted in Kuwait, Pakistan and UK, and another study conducted in Sweden also had similar findings [29]. In our study, lower percentage of patient were cigarette smokers while none of the patients had history of drinking alcohol in their life-time. Smoking and alcohol both have been reported as significant risk factors in Western and Asian countries [30]. However, studies illustrated that smoking and alcohol consumption were less significant risk factors for EC in regions with high incidence of EC, i.e. the Central Asian esophageal cancer belt [31].

In our study, drinking tea at hot temperature was associated with EC which was similar to studies that were conducted elsewhere, that demonstrated association of drinking tea at high temperature with the risk of EC (OR = 2.1–2.5) [32]. The carcinogenesis may be caused by chronic thermal damage to the esophageal mucosa.

Physical activity is the most important modifiable determinant of risk for developing various malignant disorders including colorectal, gastric, breast and endometrial cancers [33]. Several studies have shown inverse relation between EC and physical exercise [33]. Role of regular physical exercise in patients diagnosed with some common cancers in improving the survival rate had also been illustrated [34]. Another risk factor indicated by current study for developing EC was family history of cancer. A study in this regard reported that having family history of any malignancy increased the risk of getting cancer and the risk was much higher for those whose both parents were involved by cancer [35]. Healthcare policies need to be established at the national level for encouraging lifestyle modifications, dietary changes and avoidance of habits such as cigarette smoking, consumption of snuff and hot food and beverages, especially in lower socio-economical group, for possible reduction in the disease burden.

### Limitations and suggestions

Firstly, it was a descriptive study with inclusion of a small population of cases, and therefore, the findings might be biased with limited generalizability and we could not have a comparison/control group. Secondly the recall and selection are the common biases in all such studies, however, we tried our best to avoid or reduce these biases, but still these can be considered as limitation of our study. Since the current study was conducted in a single institution, therefore it was not possible to examine all the risk factors associated with development of EC. Similarly, all the biopsy samples were taken through endoscopy and tumour staging was not possible. Therefore, we suggest further large scale epidemiological studies to elaborate about all the risk factors and to explore the relationship between tumour stage and risk factors.

## Conclusion

After extensive literature review, to the best of our knowledge, the present study was found to be the first of its kind in which cases of EC diagnosis was based on histopathological examination with identification of histologic subtypes and identification of some possible risk factors for each type of EC. Majority of the patients diagnosed with EC were older males and the predominant histologic subtype was SCC. In the current study most of the cases belonged to uneducated, low-income class in which most patients were residents of rural areas Afghanistan. Similarly, most of the male patients were farmers. Considering the findings of our study, it is obvious that family history is one important risk factor that needs further elaboration.

## Electronic supplementary material

Below is the link to the electronic supplementary material.

Supplementary Material 1

## Data Availability

All data generated or analyzed during this study are included in this published article.

## Abbreviations

EC: Esophageal carcinoma
FMIC: French Medical Institute for Mothers and Children
SCC: esophageal squamous cell carcinoma
AC: esophageal adenocarcinoma
SPSS: Statistical package for social sciences
OR: Odd’s ratio
